# Severe adult ileosigmoid intussusception prolapsing from the rectum: A case report

**DOI:** 10.1186/1757-1626-1-198

**Published:** 2008-09-30

**Authors:** Rongrong Chen, Haitao Zhao, Xinting Sang, Yilei Mao, Xin Lu, Yifan Yang

**Affiliations:** 1 Department of Liver Surgery, Peking Union Medical College Hospital, Chinese Academy of Medical Sciences, CAMS & PUMC, Beijing 100730, PR China; 2 Harvard Medical School, Boston, Massachusetts, USA

## Abstract

Intussusception is a pediatric condition that rarely presents in adults. In this article, we report a case of a 36 year-old man initially presenting with abdominal pain and rectal prolapse, however, surgical reduction of the rectal prolapse did no relief his symptoms. Physical examination, abdominal plain film, barium enema and colonoscopy confirmed the presence of a large intra-abdominal mass, but the origin of the mass was revealed only upon laparotomy. During the surgery, it was noted that the ileum and the sigmoid colon was connected by a 15 cm × 12cm mass, covered by an extremely dilated intestinal tissue. The resected tissue pathology demonstrated a 9 cm × 6 cm × 5 cm submucosal lipoma at the ileocecal junction without evidence of malignancy. The patient's post-surgical course was uneventful. Diagnostic and therapeutic problems related to adult intussusception are reviewed.

## Background

Intussusception is the telescoping of one segment of the gastrointestinal tract into an adjacent one. It is usually known as a pediatric condition, found in adults with a low frequency of about 2–3 per 100,000. As the condition is relatively rare, it is often not considered in the differential diagnosis of adult patients with vague abdominal complaints. Moreover, the triad of symptoms: cramping, vomiting, and rectal bleeding are not as obvious in adults as among children, thus, making it difficult to diagnose with an even greater delay before treatment. However, as opposed to children, 90% of adult intussusceptions are associated with an identifiable etiology. About two-thirds are due to malignant tumors and with less than one-third from benign processes. Hence, there is definitely a need to identify the underlying causes of adult intussusception and provide the treatment required.

## Case presentation

**Figure 1:**
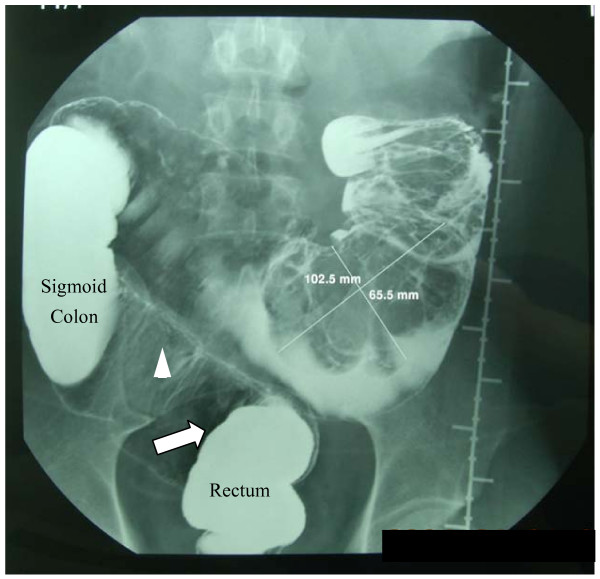
**Barium Enema The white arrow indicated the rectosigmoid junction.** Notice: (1) the irregular filling defect due to the large masses and the sharp end of the proximal colon; (2) the irregular shadow (white arrow head) in the lumen between the sigmoid colon and the rectum. It might be the prolapsed intestine which had not totally obscured the lumen as the large mass; (3) the sigmoid colon was shifted to the right abdomen.

A 36 year-old man presented to the local hospital with the chief complaint of abdominal pain and "something protruded from the rectum." He had a two-month history of diarrhoea but now had constipation for the past 2 weeks. The prolapse happened that afternoon when he was straining to defecate. Physical examination was unremarkable except for a 15 cm × 15 cm, firm, immobile, non-tender mass in the left lower quadrant of the abdomen. On rectal examination, the prolapsed was an irregular soft mass non-contiguous with the anus. An immediate surgical reduction for prolapse was performed; however, it did not relieve the abdominal pain. A subsequent barium enema (Fig 1) and a colonoscopy (not shown) revealed that the sigmoid colon was filled with a swollen intestine-like irregular mass and a separate large oval mass of about 12 cm in diameter, which prevented further visualization of the transverse or ascending colon. The patient was transferred to Peking Union Medical College Hospital for further evaluation and treatment.

**Figure 2:**
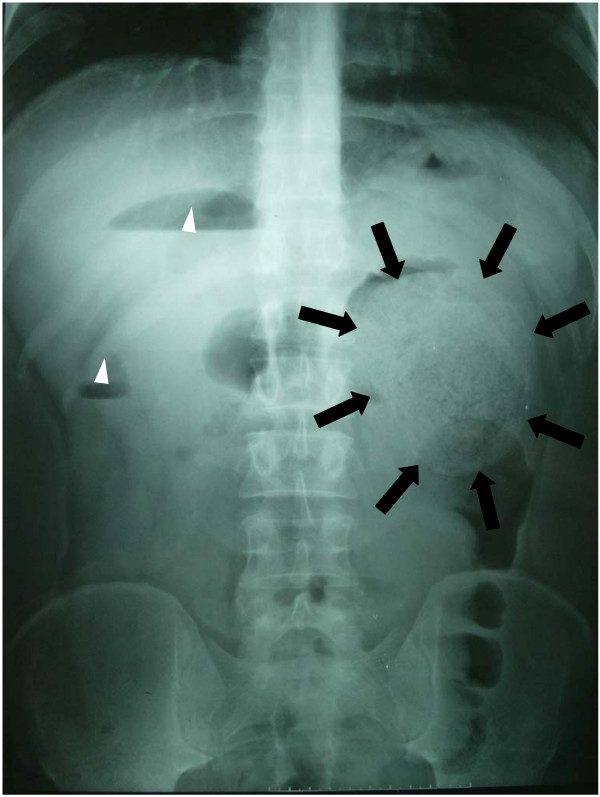
**Abdominal Plain Film.** Black arrow showed a large mass (the fecal calculus) with clear border on the left upper quarter of the abdomen. White arrow head show two vapor-liquid levels.

**Figure 3:**
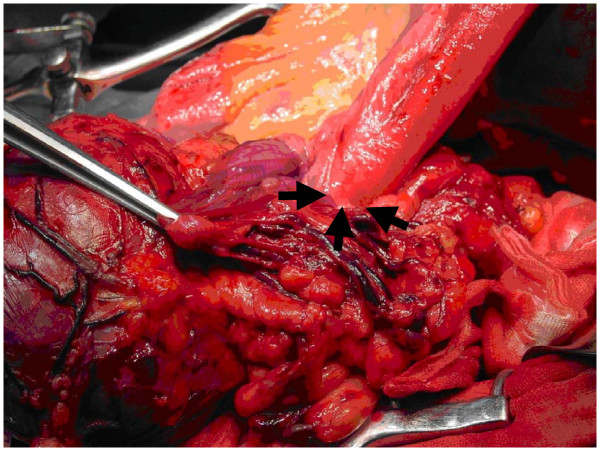
**Intussusception of the terminal ileum into the colon.** The arrows indicate the starting point of the intussusception on its rostral edge.

Upon admission, the patient still suffered from abdominal pain without symptoms of fever, tachycardia, nausea or vomiting. The abdominal plain film showed a mass with clear border on the left upper quarter (Fig 2). An exploratory laparotomy was performed. When we firstly open the greater omentum, we were astonished by what we saw – a firm oval mass about 15 cm × 12 cm located beneath the spleen instead of transverse colon (Fig 3). To figure out what the mass is, we decided to trace along the duodena, the jejunum to the distal end of the intestine, however, no ileocecal junction could be identified and the ileum and the sigmoid colon were connected by the mass. A diagnosis of extensive colonic intussusception was established. The intussusception was unable to be completely reduced by manual reduction, and a subtotal colectomy was performed.

The pathological examination demonstrated a 9 cm × 6 cm × 5 cm submucosal lipoma at the ileocecal junction without evidence of malignancy. The patient's post-surgical course was uneventful.

### Additional information

Weight: 65 kg, Height: 172 cm, no smoking, drink alcohol occasionally for business stuffs. Family history: multiple lipoma of his father and brother.

## Discussion

Intussusception occurs when a proximal segment of the bowel (intussusceptum) telescopes into an adjacent distal segment (intussuscipiens)[1]. The symptoms found in adult patients with intussusceptions are often chronic and non-specific, such as abdominal pain, fever, nausea, vomiting, melena, weight loss, and constipation. Physical examination may demonstrate diffuse or localized abdominal tenderness, while abdominal mass is detected in a minority of patients, about 24% to 42% of cases[2,3]. Gayer *et al. *suggested that computed tomography to be one of the most reliable methods of investigation for making a preoperative diagnosis of intussusception, with the classical finding being a 'target lesion' formed by a bowel-within-bowel structure, or a 'double ring' or a 'coiled spring' appearance[4,5]. Other investigations including ultrasonography, barium enema, colonoscopy, flexible sigmoidoscopy, or upper GI series, can be used according to the clinical situation, but are less sensitive and/or specific.

In this case, the patient was initially diagnosed with rectal prolapse, which should not interfere in the diagnosis of additional intussusception. In the event of a rectal prolapse, continuity may be detected through palpation between the perianal/anal tissue and the protruding tissue. In contrast, in intussusception no palpable continuity may be felt. The barium enema of the patient provided some clues to the diagnosis of intussusception such as the irregular filling defect around the large mass and the sharp end of the proximal colon, the irregular shadow in the lumen between the sigmoid colon and the rectum, which might be the prolapsed intestine. However, they were neglected. Instead, a colonoscopy instead of computed tomography was performed at the local hospital. Though this led to a number of findings important for the final diagnosis, it is still questionable whether this was a worthwhile procedure. Furthermore, the biopsy during the colonoscopy appeared unnecessary, as this patient needed a laparotomy due to the large mass occluding the colon. Though we had not performed a CT scanning before the surgery, we doubted its significance in helping with the diagnosis or surgery as the intussusception of the patient seemed to be too complicated.

**Figure 4:**
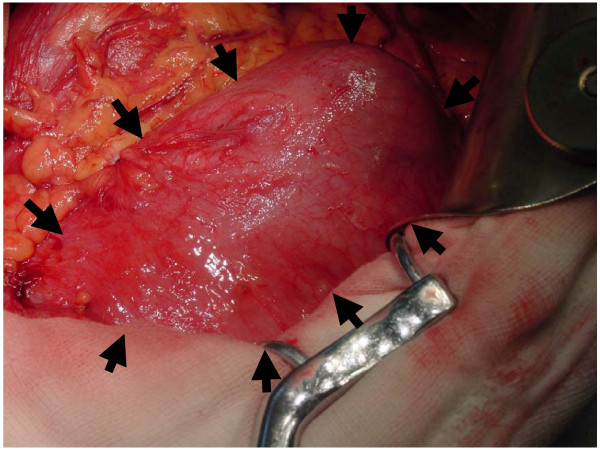
**Alvine calculus and part of the intussusception.** A submucosal lipoma located in the ileocecal junction (not shown) acts as the trigger of the intussusception, while the alvine calculus (black triangle) served as the exacerbating factor of the intussusception.

After several days of delay, he finally underwent laparotomy. On opening of the abdomen, we were astonished by what we saw. The left abdominal quadrants were occupied by a large mass covered with an extremely distended intestinal tissue (Fig 4). The intestine was disorganized appearing and normal cecum, ascending and transverse colon were absent. After careful dissociation, we finally figured out the possible formation of the rectal prolapse. The lipoma at the ileocecal junction telescoped the ileum into the ascending colon. With the colon causing dehydration, the food debris becomes thicker and harder and movement through partially occluded lumen due to the lipoma and subsequently the sigmoid colon is impeded. As more and more food debris accumulate in the proximal end of the lipoma, a fecal calculus is formed, thus come the large mass. The fecal calculus triggers a vicious circle, leading to the constipation and increased straining by the patient, which further increased the intussusception. The rectal prolapse resulted from the extremely increased intra-abdominal pressure, which causes the intestine found above the Fecal calculus to move down to the sigmoid colon (just as the barium enema showed, it was a ileosigmoid intussusception) and thru the rectum causing acute abdominal pain.

Early reports advocate reduction of adult intussusception instead of resection[6]. However, chronic intussusception do not always allow for a successful pneumatic or hydrostatic reduction to be performed, due to cross-scarring between the intussusceptum. One must also be concerned about possible malignancy, because a reduction may disseminate the malignant cells during the process. According to Begos *et al*, about 80–90% of intussusceptions in adults are secondary to an underlying pathology, with approximately 65% due to benign or malignant neoplasms[3]. However, reduction of the intussusception, especially when the small bowel is affected, maybe advantageous as it can preserve a considerable length of bowel.

In this case, after confirming benign lesion and the possible reduction, our group performed a manual reduction followed by a subtotal colectomy, allowing more ileum and sigmoid colon to be preserved. We decided that the prognostic factor involved the underlying nature of the lesion and the remaining length of functional intestine. Thus, the decision on reduction versus resection should be considered on the basis of survival and future quality of life.

## Conclusion

Adult intussusception occurs infrequently and differs from pediatric intussusception in its presentation, etiology, and treatment. Diagnosis can be delayed because of its long-lasting, intermittent, and non-specific symptoms. A more frequent use of computed tomography in the evaluation of patients with uncharacteristic abdominal pain may allow the condition to be more reliably diagnosed. Whether resection or reduction of the bowel tissue involved is still controversial. However, many speculate against reduction before resection, especially when taking into account cases where the bowel is nonviable or when malignancy is suspected. Total colectomy is not recommended if the character of the mass is not defined. Finally, exploratory laparotomy, though not the first choice for patients whose etiology remains unknown, is a necessary and sometimes effective solution.

### Learning points

• *Common symptoms such as abdominal pain, palpable masses should not be neglected in the initial assessment of patients.*

• *Adult intussusception, though rare, should be one of the differential diagnoses of vague abdominal pain, especially when found concomitantly with a palpable mass in the abdomen.*

• *Computed tomography is the cornerstone for the diagnosis of intussusception.*

• *The underlying nature of the lesion leading to the intussusception and the remnant length of functional intestine should be taken into account when one is considering treatment of adult intussusception.*

## Competing interests

The authors declare that they have no competing interests.

## Authors' contributions

RC analyzed and interpreted the patient data regarding the overall condition of the patient, and was a major contributor in writing the manuscript. HZ performed the surgery, and was also a major contributor in writing the manuscript. XS supervised the surgery and gave helpful suggestions on the surgery. YM and XL attended the surgery and contributed a lot to the writing and reviewing the manuscript. YY helped with reviewing the manuscript. All authors read and approved the final manuscript.

## Consent

Written informed consent was obtained from the patient and any accompanying images. A copy of the consent is available for review by the Editor-in-Chief of this journal.
